# The effects of *Bifidobacterium animalis* ssp. *lactis* B94 on gastrointestinal wellness in adults with Prader–Willi syndrome: study protocol for a randomized controlled trial

**DOI:** 10.1186/s13063-018-2648-x

**Published:** 2018-04-27

**Authors:** Zainab Alyousif, Jennifer L. Miller, Mariana Y. Sandoval, Chad W. MacPherson, Varuni Nagulesapillai, Wendy J. Dahl

**Affiliations:** 1 Food Science and Human Nutrition Department, UF/IFAS, University of Florida, 359 FSHN Building Newell Drive, Gainesville, FL 32611 USA; 2 Department of Pediatrics, College of Medicine, University of Florida, 2000 SW Archer Rd, Gainesville, FL 32608 USA; 3 Lallemand Health Solutions, 6100 Royalmount, Montréal, QC H4P2R2 Canada

**Keywords:** Bifidobacterium, Prader–Willi syndrome, constipation, gastrointestinal function, microbiome

## Abstract

**Background:**

Constipation is a frequent problem in adults with Prader–Willi syndrome. Certain probiotics have been shown to improve transit and gastrointestinal symptoms of adults with functional constipation. The aim of this study is to determine the effect of daily consumption of *Bifidobacterium animalis* ssp. *lactis* B94 (*B. lactis* B94) on stool frequency, stool form, and gastrointestinal symptoms in adults with Prader–Willi syndrome.

**Methods:**

Adults with Prader–Willi syndrome (18–75 years old, *n* = 36) will be recruited and enrolled in a 20-week, randomized, double-blind, placebo-controlled, crossover study. Study subjects will be randomized to *B. lactis* B94 or placebo each for a 4-week period, preceded by a 4-week baseline and followed by 4-week washouts. Subjects will complete daily records of stool frequency and stool form (a proxy of transit time). Dietary intake data also will be collected. Stools, one in each period, will be collected for exploratory microbiota analyses.

**Discussion:**

To our knowledge, this is the first randomized controlled trial evaluating the effectiveness of *B. lactis* in adults with Prader–Willi syndrome. The results of this study will provide evidence of efficacy for future clinical trials in patient populations with constipation.

**Trial registration:**

ClinicalTrials.gov (NCT03277157). Registered on 08 September 2017.

**Electronic supplementary material:**

The online version of this article (10.1186/s13063-018-2648-x) contains supplementary material, which is available to authorized users.

## Background

Prader–Willi syndrome (PWS) is a genetic disorder due to the non-expression of specific genes from the chromosome 15q11.2-q13 region [1]. The lack of imprinted genes occurs by three mechanisms, namely deletion of the 15q11.2-q13 region (found in 65–75% of individuals), maternal uniparental disomy (found in 20–30% of individuals), and an imprinting defect when the genomic region that manages the imprinting process is defective (1–3% of individuals) [1]. The estimated prevalence of PWS is 1/10,000 to 1/30,000 people [1]. At an early age, infants have severe hypotonia and feeding difficulties followed by weight gain and subsequent hyperphagia during childhood [1]. Due to altered body composition and depressed basal metabolic rate in adults with PWS [2], lifelong energy restriction is required to circumvent the development of morbid obesity [3].

Constipation is a common problem for individuals with PWS. A study of adults with PWS showed that 40% of these individuals have constipation symptoms, including having less than three defecations per week, sensation of anorectal obstruction, straining during defecation, and having hard stools [4]. Although adequate dietary fiber may be achievable in children with PWS [5], this may be a challenge in adults with PWS, particularly given their low energy needs and, thus, limited food choices. Certain probiotics, “*live microorganisms that when administered in adequate amounts confer a health benefit to the host*” [6], have been shown to improve constipation symptoms in otherwise healthy adults. Probiotics have been shown to significantly decrease transit time, increase stool frequency, and improve stool consistency, particularly *Bifidobacterium lactis* strains [7]. This study will build on previous literature reporting on the efficacy of probiotics in adults with constipation and will examine the effect of *Bifidobacterium animalis* ssp. *lactis* B94 (*B. lactis* B94) in adults with PWS. The aim of the study is to determine the effect of *B. lactis* B94 on stool frequency, stool form (a proxy for transit time), gastrointestinal (GI) symptoms, and fecal microbiota in adults with PWS. It is hypothesized that *B. lactis* B94 will increase stool frequency, decrease the percentage of slow transit stools, and improve GI symptoms. The primary outcome of the study is weekly stool frequency (difference between treatments), whereas secondary outcomes are weekly stool frequency (percentage change from baseline), stool form (percentage change in slow transit Bristol Stool Form Scale (BSFS) 1 and 2 [8] from baseline and between treatments), GI symptoms, and compliance (recorded intake of the provided and remaining supplements).

## Methods

### Design

**Fig. 1:**
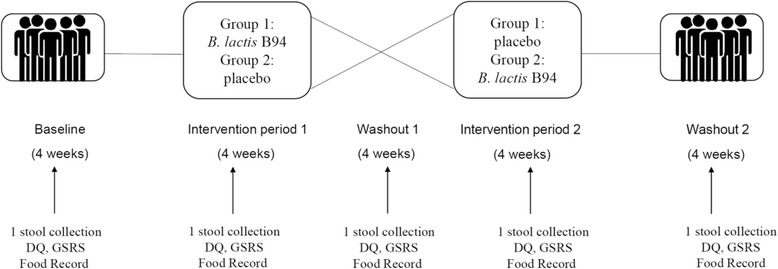
Study Design (*DQ* daily questionnaire, *GSRS* Gastrointestinal Symptom Rating Scale, *B. lactis* B94 *Bifidobacterium animalis* ssp. *lactis* B94)

A 20-week randomized, double-blinded, placebo-controlled crossover study will be carried out (Fig. 1). Adults with genetically confirmed PWS will be recruited. Subjects will complete a 4-week baseline period during which data on daily stool frequency and stool form will be collected. Dietary intake data (food record) will be obtained during the baseline period and subjects will collect a single stool. Subjects will be randomized on or about day 29 and will consume one capsule per day of *B. lactis* B94 or placebo for 4 weeks, followed by a 4-week washout, 4 weeks on the alternative treatment, and a second 4-week washout.

**Fig. 2:**
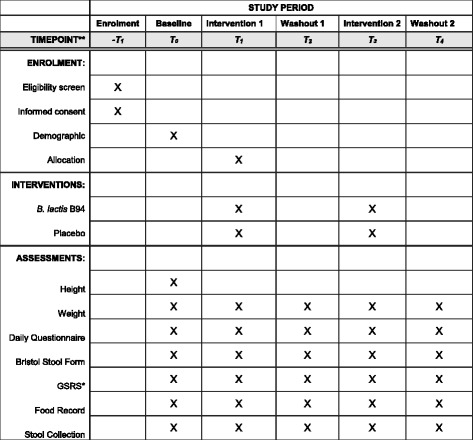
Schedule of enrollment, interventions, and assessments as per Standard Protocol Items: Recommendations for Interventional trials (SPIRIT)

During the intervention and washout periods, subjects will continue a daily record of stool frequency, stool form, and compliance. In addition, subjects will complete the Gastrointestinal Symptom Rating Scale (GSRS) [9, 10] during weeks 4, 8, 12, 16, and 20. During these same weeks, dietary intake data (food record) and single stools will be collected per period. Subject demographics, height, and weight will be taken at baseline, and body weight during weeks 8, 12, 16, and 20. Figure 2 provides the schedule of enrollment, interventions, and assessments to be completed. This paper follows the Standard Protocol Items: Recommendations for Interventional trials (SPIRIT) (Additional file 1) and the World Health Organization Trial Registration Data Set (Additional file 2).

### Randomization and blinding

A computer-generated random number sequence will be used for allocation. Randomization will be by sealed envelope method, prepared by an individual not affiliated with the study. No blocking or stratification will be performed. The principal investigator (PI) will conduct the randomization and distribution of the investigational product (IP)/placebo. Researchers, trial participants, data analysts, and all staff members at the study sites will remain blinded for the duration of the study and until the statistical analyses are completed.

### Participants

Adults with PWS, residing in residential programs and in the community at large in the state of Florida, United States, will be recruited for this study (*n* = 36). Potential subjects will be identified and contacted by their physician. The study coordinator will then meet with interested individuals, provide them with a full description of the study, and enroll those eligible. Potential subjects will be included if they have a genetically confirmed diagnosis of PWS and are 18–75 years of age, willing to have height and weight measured and to provide demographic information (e.g., age, race, sex), willing to consume *B. lactis* B94 and placebo each for a 4-week period, willing to complete a daily record of stool number and form throughout the 20-week period, willing to complete the GSRS monthly, and willing to provide information about their dietary intake every 4 weeks. Potential subjects will be excluded if they have a milk protein allergy, are currently taking medications for diarrhea or probiotics supplements and do not want to discontinue prior to the start of the baseline period (i.e., those that discontinue will be included), and were previously or are currently being treated for GI diseases, including gastric ulcers, Crohn’s disease, celiac disease, ulcerative colitis, or GI cancer. Subjects will receive their usual clinical care throughout the study.

### Intervention

Subjects will be randomized to consume either *B. lactis* B94 or placebo for a 4-week period. A 4-week supply of the *B. lactis* B94 or placebo will be given during the study visit at the start of the intervention periods. Capsules will be stored at refrigerator temperature. The IP will be *B. lactis* B94 at a dose of 15 billion colony forming units (CFU) in Veggie capsule #1 made with potato starch and magnesium stearate, and the placebo will contain potato starch and magnesium stearate (Lallemand Health Solutions Inc., Mirabel, Canada). Prior to delivery to the study site, the IP and placebo will be coded by an unblinded employee of the manufacturer. The IP and placebo will be coded with five codes each, so in the event of an adverse event that requires unblinding of the effected subject, information about most remaining subjects will remain blinded. The IP and placebo capsules will be stored in a locked refrigerator at the research site with access limited to the PI and study coordinator.

### Compliance

Study subjects will be asked to return unused capsules at the end of each intervention period to document compliance. Compliant subjects will be defined as more than 80% of supplement intake.

Recovery of *B. lactis* B94 from fecal samples will also be used to assess compliance. Study coordinators will be in frequent contact with the study participants throughout the study to promote compliance to the protocol.

### Withdrawal criteria

Subjects will be free to withdraw their consent and to stop participating in the study at any time. Subjects may be withdrawn from the study by the PI for non-compliance (e.g., do not consume the study capsules). Consent forms, given to all subjects, will specify the contact person in the case of adverse events occurring during the study. In a circumstance that a professional intervention is necessary due to an adverse event, the PI will contact the study’s affiliated physician and appropriate authority or organization pertinent to the circumstance of the event. If deemed necessary, the manufacturer of the capsules will be contacted to unblind the subject (one of 10 codes). As there are no known risks for consuming the probiotic, *B. lactis* B94, no negative side effects or adverse events are expected and, thus, no data monitoring committee is needed.

### Outcomes

#### Stool form

Stool form will be assessed using the BSFS, a simple tool for estimating intestinal transit time [8]. The BSFS classifies stools into seven categories, including type 1, separate hard lumps, like nuts; type 2, sausage-shaped, but lumpy; type 3, like a sausage but with cracks on the surface; type 4, like a sausage or snake, smooth and soft; type 5, soft blobs with clear-cut edges; type 6, fluffy pieces with ragged edges, a mushy stool; type 7, watery, no solid pieces [8]. These types are categorized into slow transit (types 1 and 2), normal transit (types 3–5), and fast transit (types 6 and 7).

#### GI symptoms

GI symptoms will be assessed using the GSRS, a 7-point scale that evaluates symptoms of GI disorders, where 1 represents no discomfort at all and 7 represents very severe discomfort over the past week [9, 10]. Symptoms of the GSRS are grouped into five GI syndromes, which are abdominal pain, reflux syndrome, diarrhea syndrome, indigestion syndrome, and constipation syndrome.

#### Microbiota analysis

Subjects will be asked to collect one stool each during the baseline, intervention 1, washout 1, intervention 2, and washout 2 periods. Subjects will be provided with Fisherbrand^®^ commode collection kits for stool collection. Samples will be processed and appropriately stored in the lab within 6 h after defecation. Stool samples will be analyzed for changes in microbiota. Changes in the concentration of *Lactobacilli* and *Bifidobacteria* genera as well as *Bifidobacterium animalis* sp. *lactis* will be measured in fecal samples by real-time PCR (qPCR).

Total DNA will be extracted from homogenized fecal samples using the QIAamp^®^ Fast DNA Stool Mini Kit (Qiagen, Hilden, Germany) as per the manufacturer’s instructions, with minor modifications, i.e., two 0.05 M phosphate buffer washes prior to the addition of InhibitEX (Qiagen) and a 1 mm zirconia/silica bead beating step (~250–350 mg/tube, 4 m/s for 1 min × 3) before centrifugation of samples to pellet stool particles. Purified DNA will be further processed for qPCR and 16S rRNA gene amplicon sequencing.

For qPCR, template DNA for standard curves will be generated by spiking feces with a bacterial suspension consisting of lyophilized bacterial powder (Lallemand Health Solutions) in HyClone phosphate buffered saline. Total cell count of each bacterial suspension will be determined by flow cytometry. Feces will be spiked with a volume equivalent to 10^9^ bacteria, and then subjected to DNA extraction as described above.

Quantification of *Lactobacilli*, *Bifidobacteria*, and *Bifidobacterium animalis* sp. *lactis* will be performed by qPCR using the ViiA™ 7 Real-Time PCR System (Thermo Fisher Scientific). Standard curves will be generated by serially diluting DNA from spiked feces. DNA samples to be quantified will be diluted in molecular biology grade water prior to qPCR.

The qPCR reaction mixture will consist of the appropriate primers, 1X SYBR^®^ Select Master Mix (Thermo Fisher Scientific), and diluted DNA. Standard curve samples will be run in duplicate and unknown samples will be tested in triplicate. Cycling conditions will consist of initial incubations, followed by 40 cycles of denaturation, annealing, and extension. A dissociation curve analysis (60°C to 95°C) will also be performed to ensure amplification specificity of the primers.

Libraries for sequencing will be prepared according to Illumina’s 16S Metagenomic Sequencing Library Preparation guidelines (Part # 15044223 Rev. B), with the exception of using Qiagen HotStar MasterMix for the first PCR (‘amplicon PCR’) and halving reagent volumes for the second PCR (‘index PCR’). As per Illumina’s guidelines, template-specific primers will target the V3-V4 region of the 16S rRNA gene (PMCID: PMC3592464) [11]. Resulting sequence reads will be analyzed through the National Research Council’s (Montreal, Canada) 16S rRNA gene amplicon analysis pipeline, as previously described [12, 13]. Reads will be QCed, paired-end assembled, and clustered at 97% similarity. Taxonomic summaries and alpha (observed) and beta (weighted or unweighted UniFrac and Bray-Curtis distances) diversity metrics, statistical analysis, and taxonomic classifications will be computed using QIIME software [14] and downstream analyses will be performed with in-house Perl and R scripts at the National Research Council.

### Sample size determination

For the power calculation, mean differences between the treatment group and the placebo group were obtained from Ishizuka et al. [15], who reported that, for constipated subjects, the mean frequency of bowel movements per week was 3.8 at baseline, 4.4 in the second week for those on placebo, and 5.1 in the second week for those on treatment. Data were simulated for each of the two experimental designs for 10,000 subjects assuming correlated Poisson random variables with mean weekly bowel movements as given above. Either the weekly totals as simulated or the averages of the weekly totals for each period (baseline, intervention) were used in subsequent analyses. For the simulations, all washouts were assumed to have the same mean as the placebo (4.4 bowel movements/week) and no temporal trend within each intervention or washout period was assumed. A correlation of 0.35 for the repeated observations on a subject was assumed. This value was obtained from previous studies involving measurement of weekly total bowel movements such as that of Baird et al. [16]. All power analyses were based on using a type I error rate of 0.05 and no correction for possible multiple comparisons. A cross-over study with five time periods, i.e., 4 weeks of baseline, 4 weeks on one treatment, 4 weeks of washout, 4 weeks on the alternative treatment, and 4 weeks post-treatment, was considered. Within each time period, the weekly totals to obtain an average total within each period for each subject were averaged. Hence, the value for any time period for a subject is the mean of the 4 weekly totals. Hypothesis testing included the F-tests in the ANOVA table for ‘Period’ and a *t* test of the difference between the product and placebo. The recommended sample size for this model is 26 to determine a difference between treatments. If the weekly means and then the treatment differences by averaging the least squares means are tested after analysis, the same sample size is required, confirming the appropriate sample size of 26. A washout of 4 weeks is expected to be sufficient to minimize any carryover effects. However, with a possibly high dropout rate of approximately 25% (due to the length of the study), a sample size of 36 is targeted. The targeted sample size is feasible as recruitment will be from a population that includes individuals participating in residential programming and those residing in the community at large.

### Statistical analysis

For the primary outcome of stool frequency, data will be analyzed using a general linear mixed model with intervention, week, period, sequence, and the interactions of period, sequence and intervention with week (weekly data), as the main fixed effects. A random effect of subject will be included in the models. Pairwise tests and post-hoc analyses will be conducted using the Tukey–Kramer method. Kenward–Roger adjustments for the denominator degrees of freedom will be performed to adjust for bias in the covariance estimates for the random effect.

### Data management

Data and files will be secured in locked cabinets and office space. Paper questionnaires will only include date and assigned participant number. Data that is originally captured as hardcopy/paper (e.g., questionnaires, etc.) will be transcribed to encrypted electronic files. Following the completion of the study, identifiers will be removed from all data.

## Discussion

In this paper, we present a clinical trial design to evaluate the effects of the probiotic *B. lactis* B94 in adults with PWS. *B. lactis* B94 may increase stool frequency, decrease the percentage of slow transit stools, and improve GI symptoms. When consuming the probiotic, subjects may experience improved bowel habits.

This study has limitations. The sample size was determined based on a study of constipated adults as no known published research has explored *B. lactis* administration in PWS subjects. As PWS is a unique patient population, their response to *B. lactis* may differ from those without such a diagnosis. A differing response is possible due to potential disease-specific changes in GI function in individuals with PWS or dissimilarities in dietary intake as individuals with PWS are commonly prescribed energy-restricted diets [3], both of which may impact baseline microbiota and response to *B. lactis*. In addition, bowel habits of individuals with PWS residing in a residential home environment may differ from adults living independently. Furthermore, given the limited population to sample, we are recruiting individuals with PWS independent of constipation status and thus, non-constipated participants may differ in their response to *B. lactis*.

To our knowledge, this is the first randomized, controlled trial evaluating the effectiveness of a *B. lactis* strain on GI wellness in adults with PWS. The results of this study will provide information on the efficacy of *B. lactis* B94 in populations at risk for slow transit and constipation. In addition to pursing publication of the findings of this trial, the authors plan to communicate the results to the study participants and healthcare professionals involved in the care of individuals with PWS.

## Trial status

The current protocol (IRB201701976, version 3) was approved December 4, 2017. Study recruitment began December 2017 and is expected to be completed by December 2018.

## Additional files

Additional file 1: SPIRIT checklist. (PDF 169 kb)

Additional file 2: World Health Organization Trial Registration Data Set. (PDF 98 kb)

## Abbreviations

BSFS: Bristol Stool Form Scale
CFU: Colony forming units
GI: gastrointestinal
GSRS: Gastrointestinal Symptom Rating Scale
IP: investigational product
PI: principal investigator
PWS: Prader–Willi syndrome
