# Supplementary material for: The effects of Bifidobacterium animalis ssp. lactis B94 on gastrointestinal wellness in adults with Prader–Willi syndrome: study protocol for a randomized controlled trial
Source: Trials. 2018 Apr 27;19:256. doi: 10.1186/s13063-018-2648-x (PMC5921784; doi:10.1186/s13063-018-2648-x)
Supplement: Supplementary file 2 — World Health Organization Trial Registration Data Set. (PDF 98 kb) [file 13063_2018_2648_MOESM2_ESM.pdf]

Data category	Information
Primary registry and trial identifying number	ClinicalTrials.gov<br>NCT03277157
Date of registration in primary registry	8 September 2017
Secondary identifying numbers	IRB201701976
Source(s) of monetary or material support	Lallemand Health Solutions Inc.
Primary sponsor	University of Florida
Secondary sponsor(s)	N/A
Contact for public queries	WD wdahl@ufl.edu
Contact for scientific queries	WD<br>University of Florida, Gainesville FL USA
Public title	B. lactis B94 effects on gastrointestinal function.
Scientific title	The effects of *Bifidobacterium animalis ssp. lactis* B94 on gastrointestinal function in adults with Prader-Willi syndrome: A randomized, double-blind study.
Countries of recruitment	United States
Health condition(s) or problem(s) studied	Gastrointestinal wellness and function
Intervention(s)	Active comparator: *Bifidobacterium animalis ssp lactis* B94
	Placebo comparator: potato starch and magnesium stearate
Key inclusion and exclusion criteria	Ages eligible for study: 18-75 years<br>Sexes eligible for study: both<br>Accepts healthy volunteers: no
	Inclusion criteria: adults with Prader Willi Syndrome (18-75 years)
	Exclusion criteria: milk protein allergy, currently taking medications for diarrhea, currently taking probiotics supplements and do not want to discontinue prior to the start of the baseline period (i.e. those that discontinue will be included), and previously or are currently being treated for gastrointestinal diseases including: gastric ulcers, Crohn's disease, celiac disease, ulcerative colitis, or gastrointestinal cancer.
Study type	Interventional
	Allocation: randomized intervention model. Crossover assignment masking: double blind (subject, caregiver, investigator, outcomes assessor)
	Primary purpose: gastrointestinal function
	N/A

Data category	Information
Date of first enrolment	Pending
Target sample size	36
Recruitment status	Recruiting
Primary outcome(s)	weekly stool frequency (difference between treatments)
Key secondary outcomes	weekly stool frequency (percentage change from baseline), stool form (percentage change in slow transit Bristol Stool Form Scale 1 and 2 from baseline and between treatments), gastrointestinal symptoms (difference between treatments and change from baseline0
